# The influence of iron nutrition on the development of intestine and immune cell divergency in neonatal pigs

**DOI:** 10.1186/s40104-024-01068-7

**Published:** 2024-08-11

**Authors:** Yao Liu, Aimin Wu, Bing Yu, Jun He, Jie Yu, Xiangbing Mao, Ping Zheng, Yuheng Luo, Junqiu Luo, Junning Pu, Hui Yan, Daiwen Chen

**Affiliations:** Key Laboratory of Animal Disease-Resistance Nutrition of China Ministry of Education, Key laboratory of Animal Disease-resistant Nutrition and Feed of China Ministry of Agriculture and Rural Affairs, Key laboratory of Animal Disease-resistant Nutrition of Sichuan Province, Institute of Animal Nutrition, Sichuan Agricultural University, Chengdu, Sichuan 611130 People’s Republic of China

**Keywords:** Immune cells, Intestine, Iron nutrition, Neonates, Redox homeostasis

## Abstract

**Background:**

Appropriate iron supplementation is essential for neonatal growth and development. However, there are few reports on the effects of iron overload on neonatal growth and immune homeostasis. Thus, the aim of this study was to investigate the effects of iron nutrition on neonatal growth and intestinal immunity by administering different levels of iron to neonatal pigs.

**Results:**

We found that iron deficiency and iron overload resulted in slow growth in neonatal pigs. Iron deficiency and iron overload led to down-regulation of jejunum intestinal barrier and antioxidant marker genes, and promoted CD8^+^ T cell differentiation in jejunum and mesenteric lymph nodes (MLN) of pigs, disrupting intestinal health. Moreover, iron levels altered serum iron and tissue iron status leading to disturbances in redox state, affecting host innate and adaptive immunity.

**Conclusions:**

These findings emphasized the effect of iron nutrition on host health and elucidated the importance of iron in regulating redox state and immunity development. This study provided valuable insights into the regulation of redox state and immune function by iron metabolism in early life, thus contributing to the development of targeted interventions and nutritional strategies to optimize iron nutrition in neonates.

## Background

Iron is an essential micronutrient that plays vital roles in the development of the immune system [1–3]. Studies have shown that iron deficiency inhibits immune cell proliferation, activation and the production of effector molecules [4–7]. Neonatal pigs are born with limited iron stores and delayed iron supplementation lead to iron deficiency, reducing the antibody response of B cells and T cells to influenza virus infection [8]. Appropriate iron supplementation has been shown to improve immune development in pigs by stimulating cytokine production and enhancing the phagocytosis of monocytes [9]. However, iron overload can lead to oxidative stress that disrupts the host immune response, increasing the risk of pathogen infection [10–14]. Despite these findings, limited studies have explored how iron homeostasis influences immune development in neonatal pigs.

Neonatal intestinal health is essential for early neonatal development. However, neonates have an underdeveloped immune system and the gastrointestinal tract is susceptible to pathogen infection, such as *Escherichia coli* (*E. coli*) and porcine epidemic diarrhea virus (PEDV) [15, 16]. It has been shown that high expression of transferrin receptor 1 in the intestinal epithelium of iron deficiency neonatal pigs promotes acute susceptibility to PEDV [17]. Iron is also an essential trace element for microbial growth and proliferation [18]. Iron overload promotes *E. coli* infection and leads to increased host cell death and increased incidence of diarrhea [19, 20]. These studies implied that iron homeostasis has been involved in the development of gastrointestinal tract, but the underlying mechanism related to the regulation of intestinal immune development in neonatal pigs still needs more research.

In this study, we established iron deficiency, normal iron and iron overload neonatal pig models by intramuscular injection of different doses of iron dextran, aiming to investigate the effects of iron nutrition on neonatal growth and development, intestinal health and immune homeostasis. Our findings showed that iron deficiency and iron overload reduced the growth performance of pigs, and disrupted redox homeostasis in neonatal pigs. Moreover, iron deficiency and iron overload reduced serum inflammatory factor levels and affected immune cell differentiation in jejunum mucosa and MLN. These results reveal a complex interaction between iron metabolism, redox homeostasis and immunity.

## Materials and methods

### Ethics statement

All procedures involving experimental animals followed the guidelines for the care and use of experimental animals established by the Ministry of Agriculture and Rural Affairs of China. This study was approved by the Animal Care and Use Committee (ACUC) at Sichuan Agricultural University Institutional Animal Care and Use Committee (SICAU-2022-10).

### Experimental animals

**Fig. 1:**
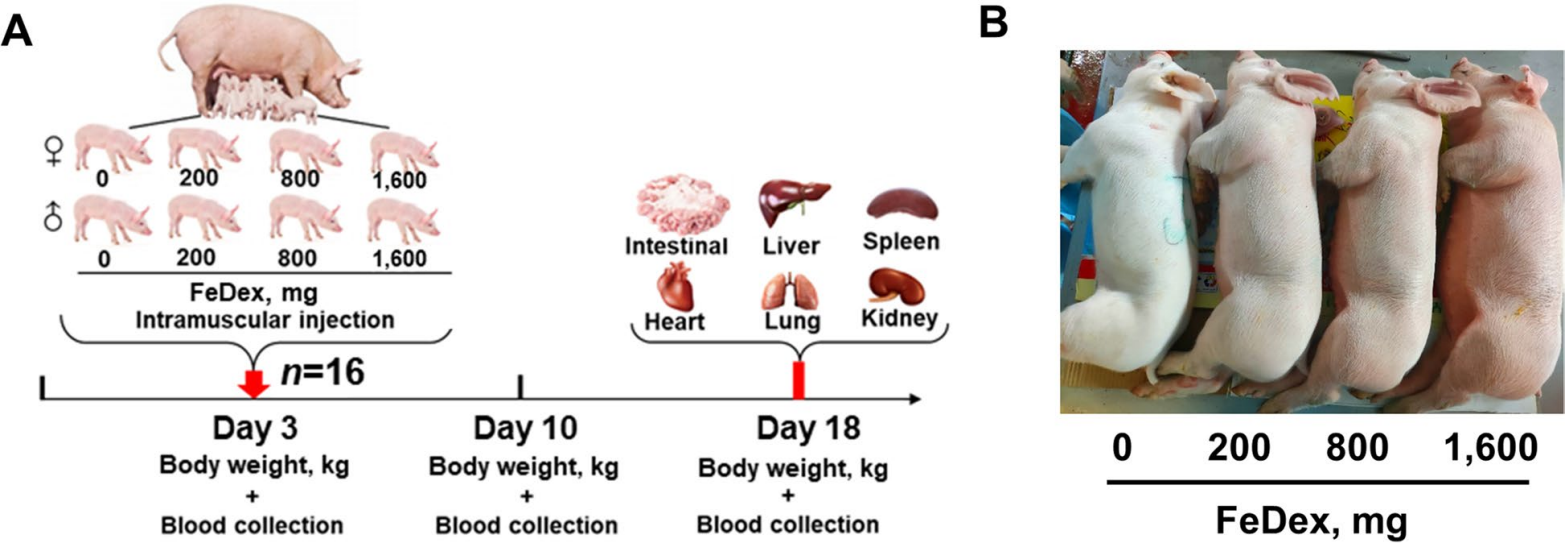
Experimental design and iron deposition. **A** The experimental design of this study. **B** Fur color of pigs with different iron levels at d 18

Sixty-four newborn Large White × Landrace × Duroc pigs (1.45 ± 0.02 kg) including 32 male pigs and 32 female pigs, were randomly assigned to 4 groups: pigs given no iron supplementation (0 mg FeDex), intramuscular injection of iron dextran (Dabeinong, China) at 200 mg Fe on d 3 (200 mg FeDex), intramuscular injection of iron dextran at 800 mg Fe on d 3 (800 mg FeDex) and intramuscular injection of iron dextran at 1,600 mg Fe on d 3 (1,600 mg FeDex) (Fig. 1A). Every fourth female and male pigs were from the same litter to exclude the effect of litter size. The pigs were raised mixed with eight lactating sows and given free access to mother milk in farrowing crates at approximately 70% humidity and a temperature of 30 ± 2 °C.

### Sample collection

Blood samples were collected from the cervical vein into EDTA vacuum tubes for immediate blood biochemical parameter analysis on d 3, 10 and 18 after birth. Serum samples were left at room temperature for 30 min, then centrifuged at 3,000 r/min for 15 min and stored at –20 °C. On d 18, the pigs were anesthetized with sodium pentobarbital solution (4%, 40 mg/kg) and euthanized for sample collection. The internal organs of the pigs, heart, liver, spleen, lungs and kidneys were taken for weighing and recording. Intermediate segments of the liver, spleen, duodenum, jejunum and cecum were taken for tissue iron assay. Jejunum and liver were fixed in 4% paraformaldehyde for morphological and histomorphology assessment. Jejunum mucosa samples were collected by using a sterile glass microscope slide, quickly frozen in liquid nitrogen, and then stored at –80 °C for further analysis.

### Determination of blood biochemical parameters

White blood cell (WBC), neutrophil count (Neu#), lymphocyte count (Lym#), monocyte count (Mon#), eosinophil count (Eos#), basophil count (Bas#), red blood cell count (RBC), hemoglobin (HGB), hematocrit (HCT), mean corpuscular volume (MCV), mean corpuscular hemoglobin (MCH) and mean corpuscular hemoglobin concentration (MCHC), platelet (PLT) and platelet hematocrit (PCT) were determined with an automatic hematology analyzer (Hitachi 3100).

### Serum and tissue iron concentrations

Serum iron was determined according to Pointe Scientific Iron/total iron binding capacity (TIBC) reagents. Specifically, a 96-well plate was prepared by adding 20 μL of dH_2_O, standard or samples. Then, 100 μL of iron buffer was added to each well and absorbance was measured at 560 nm (A1) using a spectrophotometer. Subsequently, 2 μL iron color reagent was added to each well, incubate at 37 °C for 10 min, and measure the absorbance at 560 nm (A2) using a spectrophotometer. Serum iron concentration was calculated as follows: iron (μg/dL) = (A2 sample – A1 sample)/(A2 standard – A1 standard) × concentration of standard. Unbound iron binding capacity (UIBC) (μg/dL) = concentration of standard – (A2 sample – A1 sample)/(A2 standard – A1 standard) × concentration of standard. TIBC (μg/dL) = serum iron + UIBC. Transferrin saturation (TS) (%) = Serum iron/TIBC × 100%.

Tissue non-heme iron content was determined by specific experimental methods. Specifically, 0.1 g of tissue sample was digested in 1 mL of tissue digest at 65 °C for 72 h. After digestion, the digest was fixed to 1.5 mL. A 96-well plate was prepared by adding 10 μL of dH_2_O, standard or samples. Then, 200 μL of iron color reagent was added to each well, and the absorbance was read at 535 nm (OD_535nm_) after 10 min of incubation at room temperature. Tissue iron content was calculated as follows: tissue iron (g/g) = OD_535nm_/tissue weight × (1.5 – 0.25 × tissue weight) × (1 × iron standard OD_535nm_ × 4.77).

### Reverse transcription and quantitative real-time PCR

Following the manufacturer’s instructions, RNA samples were extracted from tissues using the TRIzol reagent (Invitrogen) and normalized to 1 μg/μL. The reaction solution was configured in accordance with the Reverse Transcription Kit’s instructions (Takara). Using the ΔΔCT approach, the relative gene expression was computed; the data were then normalized to the housekeeping gene *β-actin*. Primer design is shown in Table 1.

**Table 1:** qPCR primer sequences

Gene	Forward (5′→3′)	Reverse (5′→3′)
*MUC1*	GTGCCGCTGCCCACAACCTG	AGCCGGGTACCCCAGACCCA
*MUC2*	GGTCATGCTGGAGCTGGACAGT	TGCCTCCTCGGGGTCGTCAC
*Occludin*	CTACTCGTCCAACGGGAAAG	ACGCCTCCAAGTTACCACTG
*ZO-1*	TGGCATTATTCGCCTTCATAC	AGCCTCATTCGCATTGTTT
*Claudin-1*	GCCACAGCAAGGTATGGTAAC	AGTAGGGCACCTCCCAGAAG
*SOD1*	GAGCTGAAGGGAGAGAAGACAGT	GCACTGGTACAGCCTTGTGTAT
*SOD2*	CTGGACAAATCTGAGCCCTAAC	GACGGATACAGCGGTCAACT
*GPX4*	TACGTGTGCATCGTCACCAA	TTGCAAGGGAAGGCCAGAAT
*CAT*	TGTACCCGCTATTCTGGGGA	TCACACAGGCGTTTCCTCTC
*β-actin*	TCTGGCACCACACCTTCT	TGATCTGGGTCATCTTCTCAC

### Tissue morphology and iron deposition

Intestinal and liver tissues were cut and embedded in paraffin using a Rotary Microtome (Leica, Germany), stained with hematoxylin–eosin or Prussian blue, and fixed on glass slides. The slides were observed, and villus height and crypt depth were measured using an optical microscope (Leica). At least ten visual fields were selected in each slice, and eight pairs of villi (μm) and crypts (μm) were measured in each microscopic field. The mean values were determined and calculated for the final values.

### Serum inflammatory cytokines and immunoglobulin detection

Serum concentrations of inflammatory cytokines (IL-1β, IFN-γ and TNF-α) and immunoglobulins (IgA, IgM and IgG) were determined according to the manufacturer’s (Jiangsu Meimian Industrial Co., Ltd., Jiangsu, China) instructions.

### Serum antioxidant capacity detection

Total superoxide dismutase (T-SOD), catalase (CAT), total antioxidant capacity (T-AOC), glutathione peroxidase (GHS-Px) and activities of malondialdehyde (MDA) levels in the serum were analyzed using assay kits (Nanjing Jiancheng Bioengineering Institute, Nanjing, China), according to the manufacturer’s instructions.

### Measurement of intestinal ROS

To assess intestinal ROS, the jejunum first needs to be prepared as single cell suspension. Specifically, jejunum tissue was diced and placed in 10 mL of Hanks’ balanced salt solution-dithiothreitol, and allowed to stand for 30 min at 37 °C. The supernatant was then poured out and 10 mL of Hanks' balanced salt solution—ethylenediaminetetraacetic acid was added to resuspend the jejunum tissue and shaken for 30 min at 37 °C. The supernatant was poured and 1 mg/mL of collagenase A and 1 mg/mL of DNase were added to the jejunum tissue and digested for 1 h at 37 °C. After digestion, filter the cell suspension through 70 μm cell strainer for later use. A portion of the above jejunum cell suspension was treated with 50 μmol/L H2DCFDA (Sigma-Aldrich, D6883) and 5 μmol/L CD11-BODIPY625 (Invitrogen, D3861), respectively, at 37 °C for 30 min. This was followed by 3 PBS washes. Cells were collected with a BD FACSVerse™ instrument (BD Biosciences) and analyzed with FlowJo10 software.

### Flow cytometry analysis

Single cell suspensions were labeled with APC anti-pig CD45, PE/Cy7 anti-pig CD3, PerCP/Cy5.5 anti-pig CD4a and PE anti-pig CD8a for T cells, and purified anti-mouse CD16/32, FITC anti-mouse CD45, PerCP/Cy^TM^5.5 rat anti-CD11b, APC anti-mouse CD206 and APC/Cy7 anti-mouse CD86 for macrophages. Cells were collected with a BD FACSVerse™ instrument (BD Biosciences) and analyzed with FlowJo10 software. Antibody information is listed in Table 2.

**Table 2:** Antibody information

Antibodies	Source	Identifier
Alexa Fluor^®^647 Mouse anti pig CD45	BIO-RAD	MCA1222A647
PE-Cy™7 Mouse anti-pig CD3	BD Pharmingen™	Cat # 561477
PerCP-Cy™5.5 Mouse anti-pig CD4a	BD Pharmingen™	Cat # 565853
PE Mouse anti-pig CD8a	BD Pharmingen™	Cat # 559584
Purified Rat anti-mouse CD16/CD32	BD Pharmingen™	Cat # 553141
FITC Rat anti-mouse CD45	BD Pharmingen™	Cat # 553080
PerCP-Cy™5.5 rat anti-CD11b (M1/70)	BD Pharmingen™	Cat # 550993
Alexa Fluor^®^647 rat anti-mouse CD206	BD Pharmingen™	Cat # 565250
APC/Cyanine7 anti-mouse CD86	BD Pharmingen™	Cat # 105030

### Statistical analysis

The Shampiro-Wilk test was performed prior to statistical analysis to determine normality of variance. Statistical analyses were performed by one-way ANOVA using SPSS 22.0 software (SPSS Inc., Chicago, IL, USA), with each pig as an experimental unit. All results are expressed as mean ± standard error of the mean (SEM), *P* < 0.05 deemed statistically significant. Graphs were generated using Adobe Illustrator 2021 (Adobe Incorporated, San Jose, CA, USA).

## Results

###  Iron deficiency and iron overload inhibit neonatal growth and development

We observed that a dose-dependent increase in skin color, with darker skin color implying greater iron deposition in body (Fig. 1B). 200 mg FeDex had a higher body weight than 0 mg FeDex, 800 mg FeDex and 1,600 mg FeDex at d 18 (Table 3). Based on the body weight results, we defined 0 mg FeDex as iron deficiency and 800 mg FeDex and 1,600 mg FeDex as iron overload. We also examined the organ index of neonatal pigs and found that 200 mg FeDex had a higher liver and spleen indices than 0 mg FeDex and 800 mg FeDex, whereas the heart index decreased with iron dosage (Table 3). These results suggested that iron levels affect the overall growth and development of neonatal pigs.

**Table 3:** Effects of different doses of iron dextran on growth performance and organ index of neonatal pigs

Item	FeDex, mg				SEM^1^	*P* value
	0	200	800	1,600		
Body weight, kg						
d 3	1.45	1.45	1.45	1.45	0.16	0.99
d 10	2.67	2.92	2.77	2.7	0.44	0.39
d 18	3.89^b^	4.42^a^	4.08^ab^	4.07^ab^	0.67	0.04
Average daily gain, kg/d						
d 3 to 18	0.16	0.2	0.17	0.18	0.04	0.16
Organ index, g/kg						
Liver	99.48^b^	121.93^a^	118.49^a^	119.84^a^	18.68	< 0.01
Spleen	7.16^b^	9.36^a^	8.19^ab^	9.10^a^	2.01	0.01
Heart	30.54^a^	28.84^ab^	25.24^b^	26.58^ab^	5.33	0.03
Lung	54.24	60.54	56.16	55.64	9.32	0.29
Kidney	26.53	29.83	28.48	28.82	4.75	0.27

^1^*SEM* Standard error of the means

^a,b^Values in the same row with different superscripts letters are significantly different (*P* < 0.05)

###  Iron levels affect systemic iron homeostasis and blood biochemical

**Fig. 2:**
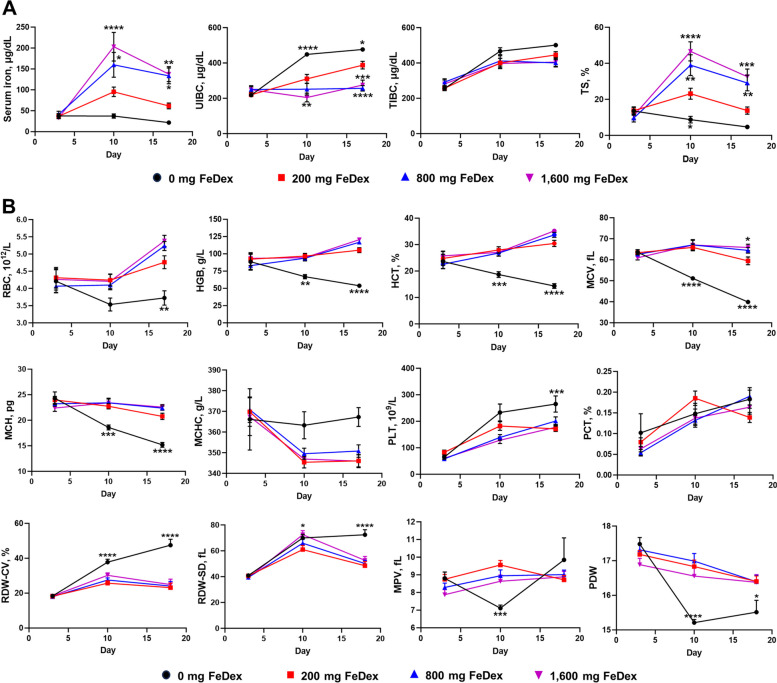
Iron levels affect iron metabolism. **A** Changes in serum iron, UIBC, TIBC and TS% from d 3 to 18. **B** Changes in RBC, HGB, HCT, MCV, MCH, MCHC, PLT, PCT, RDW-CV, RDW-SD, MPV and PDW from d 3 to 18. Data are presented as mean ± SEM and one-way ANOVA was performed. ^*^P < 0.05, ^**^P < 0.01, ^***^P < 0.001 and ^****^P < 0.0001

**Fig. 3:**
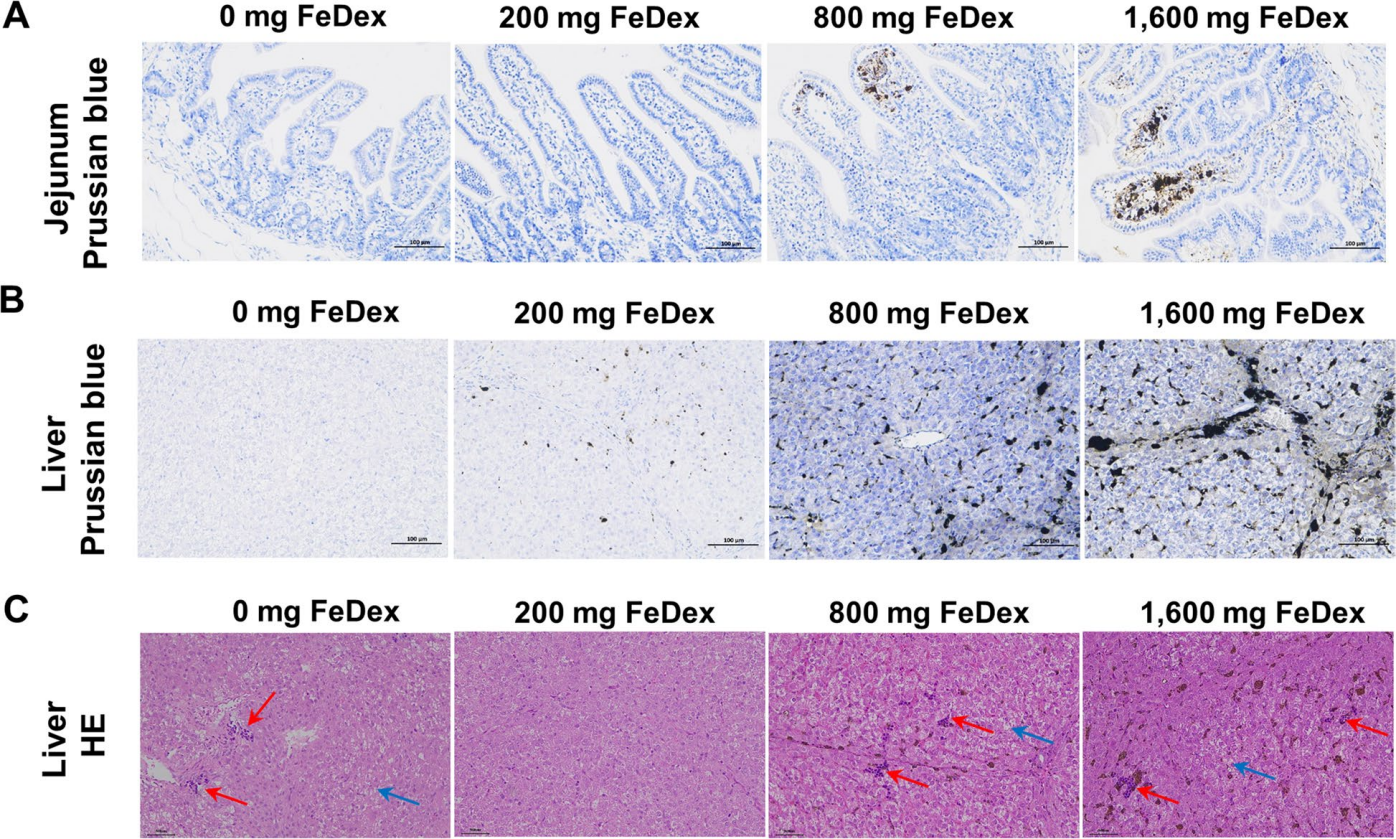
Effects of different doses of iron dextran on iron deposition and histology in neonatal pigs. **A** Prussian blue staining of jejunum. **B** Prussian blue staining of liver. **C** Hematoxylin–eosin staining of liver (Single black arrowheads: lymphocyte infiltration and blue arrowheads: round vacuoles in cytoplasm)

To assess the effect of iron levels on blood iron metabolism, we measured serum iron, UIBC, TIBC and TS. The results showed that serum iron concentration and TS increased and UIBC decreased in 800 mg FeDex and 1,600 mg FeDex at d 18 (Fig. 2A). Analysis of blood cell parameters revealed that 0 mg FeDex had significantly decreased in RBC, HGB, HCT, MCV and mean MCH compared with 200 mg FeDex, indicating that iron deficiency contributed to anemia (IDA) (Fig. 2B). Additionally, we examined the deposition of iron in tissues and showed that different tissues have different abilities to deposit iron. The iron concentrations in liver, spleen, duodenum, jejunum and cecum of pigs treated with 1,600 mg FeDex were 13%, 83%, 46%, 45% and 20% higher than those of pigs treated with 800 mg FeDex, respectively (Table 4). This indicated that the liver and cecum gradually reach saturation in iron deposition, while the spleen, duodenum and jejunum exhibit stronger iron deposition capabilities. In addition, prussian blue staining revealed that iron levels in liver and jejunum increased with iron dosage (Fig. 3A and B), and iron deficiency and iron overload resulted in infiltration of immune cells as well as vacuolization of hepatocytes (Fig. 3C). Overall, iron deposition in liver, spleen, duodenum, jejunum and cecum of pigs increased with iron dosage and excess iron caused tissue damage.

**Table 4:** Effects of different doses of iron dextran on tissue iron deposition of neonatal pigs at d 18

Item	FeDex, mg				SEM^1^	*P* value
	0	200	800	1,600		
Iron concentration, μg/g						
Liver	68.94^d^	215.03^c^	1,214.40^b^	1,372.90^a^	126.13	< 0.01
Spleen	258.20^c^	432.50^c^	1521.60^b^	2,788.40^a^	376.94	< 0.01
Duodenum	54.17^c^	61.87^c^	86.85^b^	126.74^a^	17.32	< 0.01
Jejunum	60.52^c^	66.15^c^	100.12^b^	145.20^a^	23.47	< 0.01
Cecum	78.18^c^	111.61^b^	124.62^b^	149.87^a^	28.82	< 0.01

^1^*SEM* Standard error of the means

^a−d^Values in the same row with different superscripts letters are significantly different (*P* < 0.05)

###  Iron deficiency and iron overload induce oxidative stress detrimental to intestinal health

**Fig. 4:**
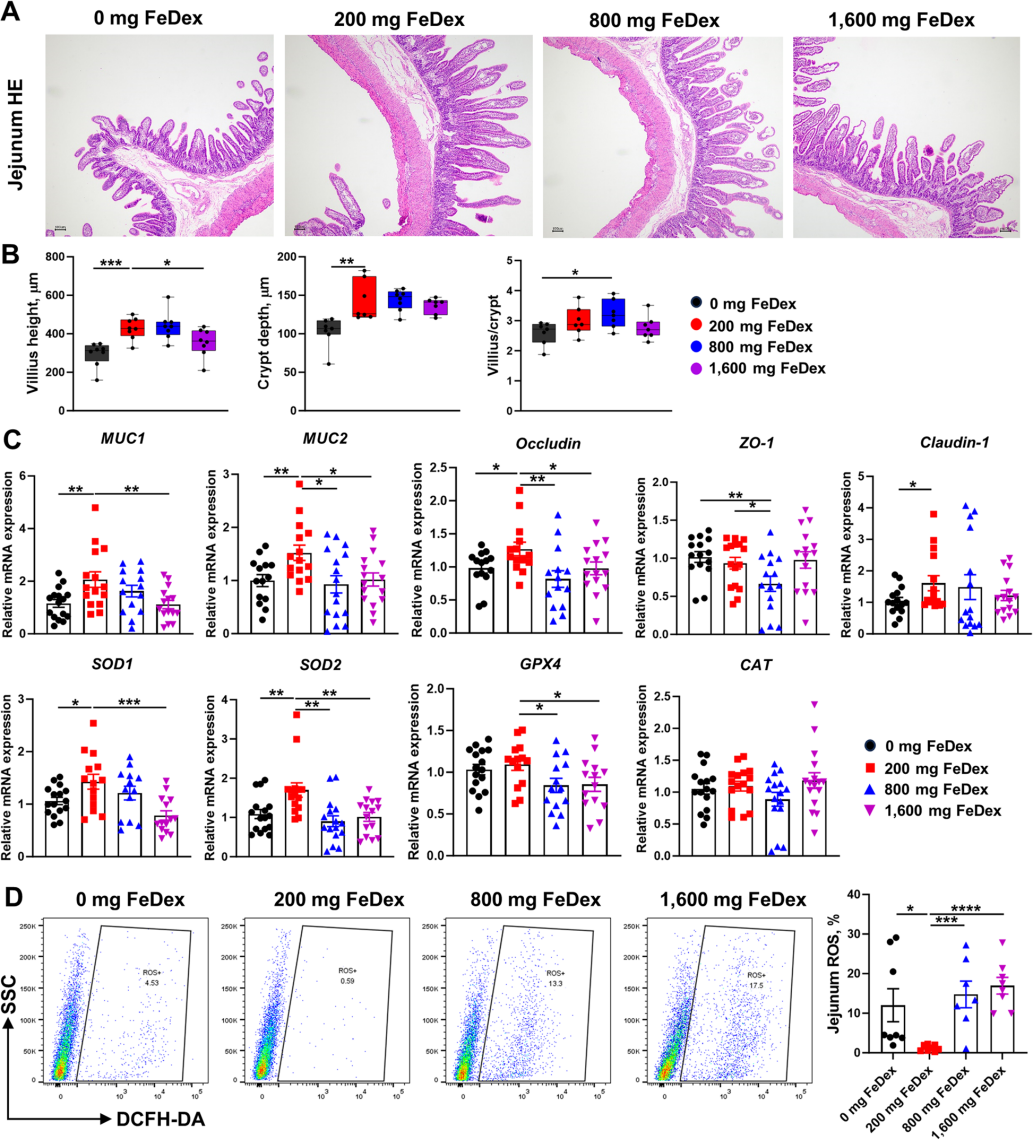
Iron deficiency and excessive iron induce oxidative stress detrimental to intestinal health. **A** Jejunum mucosa epithelium from each group were processed for morphological evaluation. **B** The relative villus height, crypt depth and villus/crypt were measured (scale bar = 100 μm). **C** mRNA expression of *MUC1*, *MUC2*, *Occludin*, *ZO-1*, *Claudin-1*, *SOD1*, *SOD2*, *GPX4* and *CAT*, measured by RT-PCR in jejunum mucosa. **D** ROS levels were detected in jejunum. Data are presented as mean ± SEM and one-way ANOVA was performed. ^*^P < 0.05, ^**^P < 0.01, ^***^P < 0.001 and ^****^P < 0.0001

To assess the effects of iron on neonatal pig intestinal health, we examined jejunum morphology in pigs injected with different doses of iron (Fig. 4A). 200 mg FeDex exhibited higher jejunum villus height, crypt depth and villus/crypt ratio compared to 0 mg FeDex, 800 mg FeDex and 1,600 mg FeDex (Fig. 4B). Additionally, 200 mg FeDex showed increased mRNA expression of genes associated with intestinal barriers (*MUC1*, *MUC2*, *Occludin* and *Claudin-1*) and antioxidant enzymes (*SOD1*, *SOD2* and *GPX4*) compared to 0 mg FeDex, 800 mg FeDex and 1,600 mg FeDex (Fig. 4C). We also examined ROS levels in jejunum mucosa and found that 0 mg FeDex, 800 mg FeDex and 1,600 mg FeDex exhibited increased ROS levels in jejunum mucosa, indicating disruption of iron homeostasis affects redox state (Fig. 4D). Consistent with the jejunum mucosa, 200 mg FeDex displayed higher antioxidant enzyme activities (T-SOD, CAT, GSH-Px) compared to 800 mg FeDex and 1,600 mg FeDex, and lower levels of MDA compared to 0 mg FeDex, 800 mg FeDex and 1,600 mg FeDex in serum (Table 5). These results indicated that iron deficiency and high iron disrupt intestinal redox state detrimental to intestinal development in neonatal pigs.

**Table 5:** Effects of different doses of iron dextran on anti-oxidative capacity in serum of neonatal pigs at d 18

Item	FeDex, mg				SEM	*P* value
	0	200	800	1,600		
T-SOD, U/mL	84.26^a^	88.20^a^	79.25^b^	76.67^b^	5.61	< 0.01
CAT, U/mL	18.51^b^	29.07^a^	19.73^b^	17.34^b^	11.82	0.03
T-AOC, mmol/L	0.44	0.48	0.42	0.37	0.11	0.09
GSH-Px, μmol/L	293.20^a^	294.03^a^	284.24^ab^	276.06^b^	18.14	0.02
MDA, nmol/mL	5.74^b^	5.61^b^	6.35^ab^	6.64^a^	1.09	0.03

*T-SOD* Total superoxide dismutase, *CAT* Catalase, *T-AOC* Total antioxidant capacity, *GSH-Px* Glutathione peroxidase, *MDA* Malondialdehyde

^1^*SEM* Standard error of the means

^a,b^Values in the same row with different superscripts letters are significantly different (*P* < 0.05)

### Iron levels affect systemic immune homeostasis

**Fig. 5:**
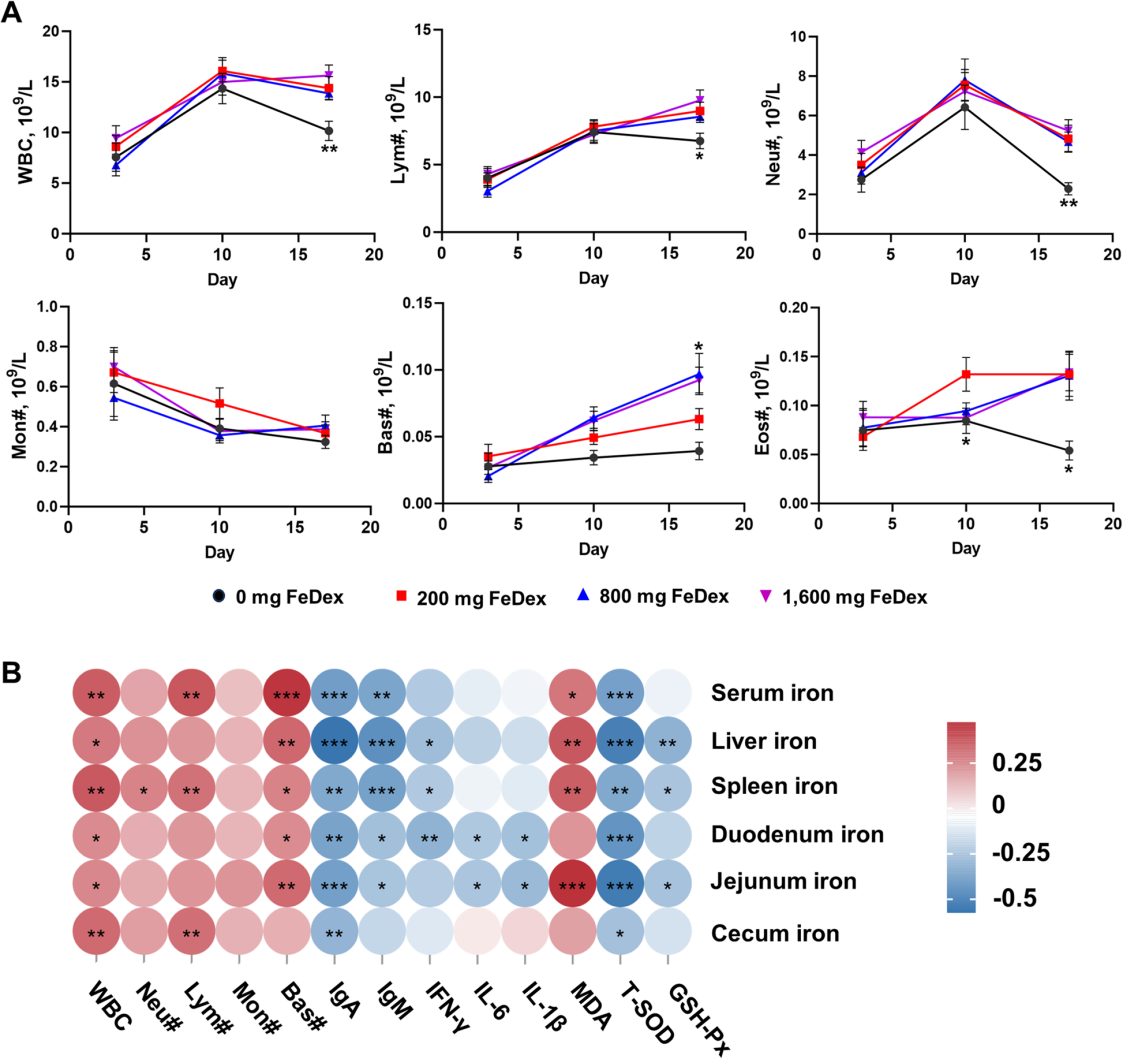
Iron levels affect host immunity. **A** Changes in blood WBC, Neu#, Lym#, Mon#, Eos# and Bas# from d 3 to 18. **B** Correlation analysis of serum and tissue iron with blood immune cells, serum immunoglobulins, inflammatory factors and antioxidant indices. Data are presented as mean ± SEM and one-way ANOVA was performed. ^*^P < 0.05, ^**^P < 0.01, ^***^P < 0.001 and ^****^P < 0.0001

To investigate the effect of iron homeostasis on host immunity, we examined the number of immune cells in blood. On d 18, 0 mg FeDex exhibited a significant decrease in WBC, Neu#, Lym# and Eos#, while 800 mg FeDex showed a significant increase in Bas# in blood (Fig. 5A). 0 mg FeDex and 200 mg FeDex showed higher levels of serum IgA and IgM compared to 800 mg FeDex and 1,600 mg FeDex (Table 6). Moreover, 200 mg FeDex group exhibited higher levels of serum TNF-α and IL-6 compared to 0 mg FeDex, 800 mg FeDex and 1,600 mg FeDex (Table 6). Correlation analysis of serum and tissue iron with immune-related and antioxidant indices in blood showed that serum iron was positively correlated with WBC, Lym#, Bas# and MDA, and negatively correlated with IgA, IgM and T-SOD (Fig. 5B).

**Table 6:** Effects of different doses of iron dextran on immunoglobulins and inflammatory cytokines in serum of neonatal pigs at d 18

Item	FeDex, mg				SEM	*P* value
	0	200	800	1,600		
IgA, μg/mL	23.21^a^	22.18^ab^	19.24^c^	20.68^bc^	2.00	< 0.01
IgM, μg/mL	27.59^a^	27.72^a^	22.67^b^	23.14^b^	4.20	< 0.01
IgG, μg/mL	337.65	336.07	339.99	348.21	32.91	0.76
TNF-α, pg/mL	49.08^b^	54.76^a^	48.18^b^	47.43^b^	5.91	< 0.01
IFN-γ, pg/mL	191.96	201.04	180.38	176.45	28.74	0.07
IL-6, ng/L	101.90^ab^	112.81^a^	103.27^ab^	97.11^b^	15.76	< 0.05
IL-1β, ng/L	4.79	5.19	4.88	4.59	0.66	0.09

*Ig* Immunoglobulin, *TNF-α* Tumor necrosis factor-α, *IFN-γ* Interferon-γ, *IL* Interleukin

^1^*SEM* Standard error of the means

^a−c^Values in the same row with different superscripts letters are significantly different (*P* < 0.05)

###  Iron levels affect the populations of T lymphocytes and macrophages in jejunum and MLN

**Fig. 6:**
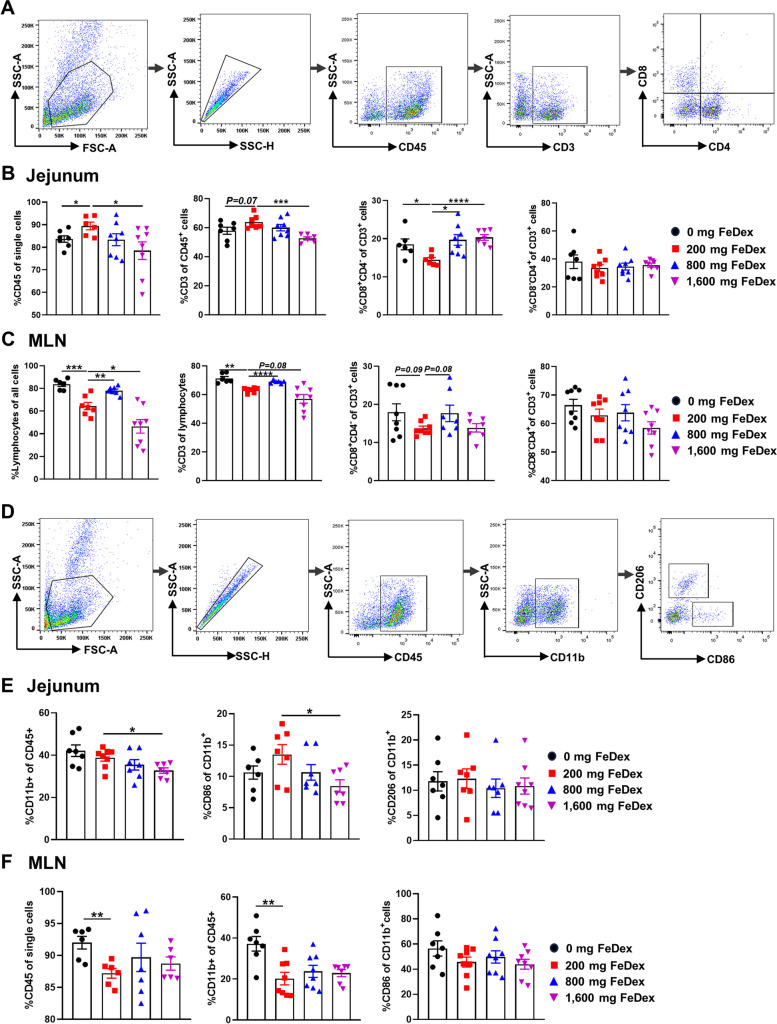
Iron levels affect jejunum and MLN immune cell populations. **A** Gating strategy for T cell flow cytometry in jejunum and MLN. Percentages of CD45^+^, CD3^+^, CD8^+^CD4^−^ and CD8^−^CD4^+^ detected by flow cytometry in jejunum (**B**) and MLN (**C**). **D** Gating strategy for macrophage flow cytometry in jejunum and MLN. Percentages of lymphocytes, CD45^+^, CD11b^−^, CD86^+^ and CD206^+^ detected by flow cytometry in jejunum (**E**) and MLN (**F**). Data are presented as mean ± SEM and student’s *t*-test was performed. ^*^P < 0.05, ^**^P < 0.01, ^***^P < 0.001 and ^****^P < 0.0001

To further investigate the regulation of intestinal immunity by iron levels, we examined immune cell subsets in jejunum and MLN. The flow chart showed gating strategy of T lymphocytes and we analyzed CD45^+^ cells and CD3^+^ cell subpopulations including CD8^−^CD4^+^ T helper cells and CD8^+^CD4^−^ cytotoxic T cells in jejunum mucosa and MLN (Fig. 6A). It was found that 0 mg FeDex and 1,600 mg FeDex significantly reduced CD45^+^ cells and CD3^+^ cells in jejunum mucosa compared to 200 mg FeDex. In addition, 0 mg FeDex, 800 mg FeDex and 1,600 mg FeDex had an increased populations of CD8^+^CD4^−^ T cell in jejunum mucosa compared to 200 mg FeDex, while CD8^−^CD4^+^ did not change significantly (Fig. 6B). MLN is an important site of T-cell activation for small intestine. Therefore, we also examined immune cell subsets in MLN. The results revealed that 0 mg FeDex and 800 mg FeDex increased lymphocytes, CD3^+^ T cells and CD8^+^CD4^−^ T cells in MLN, suggesting that iron deficiency and iron overload promoted T-lymphocyte activation and facilitated the differentiation of CD8^+^ T cells in MLN (Fig. 6C). Macrophages are critical in intestinal tissue homeostasis and host defense. The flow chart showed gating strategy of macrophage in jejunum mucosa and MLN (Fig. 6D). The results revealed 1,600 mg FeDex had a reduction of macrophages and CD86^+^ in jejunum compared to 200 mg FeDex, indicating that iron overload inhibited the polarization of M1-type macrophages (Fig. 5E). In MLN, 0 mg FeDex increased the number of macrophages compared to 200 mg FeDex, suggesting that iron deficiency promoted an increase in macrophage population (Fig. 5F).

## Discussion

Iron is a crucial micronutrient required for the growth and development of neonatal. Untimely iron supplementation of neonatal pigs after birth leads to a decrease in serum iron levels promoting the occurrence and development of IDA, including a significant decrease in hematological parameters such as RBC, HGB and HCT [21]. In this study, 0 mg FeDex neonatal pigs showed significant decreases in serum iron, HGB and HCT indicating the development of iron deficiency anemia. Appropriate iron supplementation improved anemia symptoms and increased growth of neonatal pigs. Consistent with our results that iron-deficient neonatal pigs grew slowly, iron supplementation increased neonatal weight at weaning and alleviated anemia symptoms [22]. High iron (800 mg FeDex and 1,600 mg FeDex) improved hematological indices and alleviated anemia, but reduced growth in neonatal pigs. In addition, iron overload potentiated iron hepatotoxicity, accompanied with hepatic steatosis as well as deregulated iron and bile acids homeostasis, resulting in slow growth in mice [23]. Generally, appropriate iron contributes to the growth and development of neonatal pigs.

Different organs/tissues exhibited diverse capacities for iron storage and distinct tolerance for iron levels. In this study, the liver and spleen iron levels of 1,600 mg FeDex were up to 6 times higher than those of 200 mg FeDex, proportional to the amount of iron inclusion, suggesting liver and spleen have a high storage capacity for iron. Our study supported that excess iron was mainly stored in liver and spleen [24]. However, the extra iron accumulation in liver was reported to induce ferroptosis and subsequent liver damage and fibrosis [25]. Iron overload caused free radical accumulation, and further contributed to oxidative stress and inflammation [26]. Interestedly, we also noticed that 1,600 mg FeDex only had 2 times more intestinal and serum iron levels than 200 mg FeDex, suggesting that the intestines and blood have limited capacity to store iron and can easily reach saturation, or have stronger turnover capacity. Intestines were sensitive to iron levels and have low tolerance to either iron deficiency or overload [27, 28]. Iron deficiency was reported to exacerbate DSS-induced acute colitis in mice [29]. Besides iron overload caused intestinal damage via the distribution of redox and immune homeostasis, excessive iron also contributes to dysbiosis of gut microbial, leading to an increase in harmful bacteria such as *Salmonella* and *E. coli* detrimental to intestinal health [26, 30–32].

Early intestinal development is a critical for animal health and growth. The morphology (villus and crypt) and function of intestines change rapidly during the first two weeks of age in neonatal pigs [33]. In our study, either iron deficiency or overload led to a decrease in villus height, crypt depth and villus height/crypt depth ratio in jejunum. Impaired development of intestinal villi hinders the proliferation and renewal of epithelial cells, which affects the digestive and absorptive capacity of the organism and leads to slow growth of neonatal pigs [34]. In this study, iron deficiency and iron overload resulted in downregulation of the tight junction proteins Occludin and Claudin-1 as well as the mucosal proteins MUC1 and MUC2 in jejunum mucosa, which may contribute to a greater susceptibility of neonates to pathogen infection. Research reports that there is a positive correlation between gut integrity and antioxidant capacity [35]. In this study, serum T-SOD and CAT activities were significantly decreased. Iron overload causes Fenton reaction to produce ROS. Accumulation of large amounts of iron lead to increased levels of cellular and mitochondrial ROS, which interferes with mitochondrial function [36]. Studies have reported that excessive dietary iron intake induced hepatic iron accumulation and lipid peroxidation, which inhibited *GPX4* expression through suppression NRF2-ARE pathway thereby promoting mitochondrial oxidative stress [37]. In our study, ROS levels were significantly increased in jejunum cells of both iron-deficient and iron-overloaded pigs. In addition, antioxidant genes such as *SOD* and *GPX4* were significantly reduced in jejunum mucosa of iron-deficient and iron-overloaded pigs. In summary, appropriate iron levels are essential for maintaining animal health.

Iron affects the development and function of immune cells and is strongly associated with infections [5]. Leukocytes including neutrophils, lymphocytes and eosinophils were reduced in the blood of iron-deficient pigs. Similar results were reported in mice that iron deficiency inhibits the generation of mature neutrophils in the bone marrow, leading to a significant decrease in circulating neutrophils and eosinophils [38]. These findings suggest that iron deficiency suppresses the development of immune cell populations. In this study, iron deficiency also decreased the levels of inflammatory factors such as TNF-α, IFN-γ, IL-6 and IL-1β in the serum of neonatal pigs. Recent research has shown that iron deficiency enhances mitoROS production and mitoROS-dependent NETosis, impairing the functional efficacy of neutrophils in plasma and suppressing the production of inflammatory cytokines TNF-α, IL-6, and IL-12 [38]. Additionally, iron deficiency can inhibit cyclin E1 induction and S-phase entry after B-cell activation leading to low IgG and IgM production in iron-deficience mice [39]. However, IgA, IgM and IgG levels in iron-deficient pig serum did not change in this study, which may be due to the fact that short duration of iron deficiency in pigs and did not cause damage to humoral immunity. Supplementing iron can alleviate the immunodeficiency caused by iron deficiency, but the effects of iron overload on host immunity remain unclear. In our study, iron overload, similar to normal iron supplemented pigs, alleviated the suppression of immune cell production caused by iron deficiency. However, similar to iron deficiency, iron overload suppressed inflammatory factor production. In a study, iron overload (200 μmol/L ferric ammonium citrate) inhibited the production of TNF-α and IL-1β in macrophages in response to LPS stimulation, suggesting that iron overload impairs host immune function [13]. In addition, iron overload reduced the levels of serum IgA and IgM. Similar to our results, serum IgA in weaned pigs decreased with increasing levels of dietary iron, and IgM also tended to decrease [24]. This was also observed in children with β-thalassemia, suggesting that iron overload impairs humoral immunity [40]. Collectively, iron deficiency inhibits host immune development, and iron supplementation can alleviate immunodeficiency caused by iron deficiency, but excessive iron supplementation can also impair host immunity.

Unabsorbed iron in intestinal can stimulate the proliferation and virulence of pathogens increasing the risk of infection in neonatal pigs. In recent studies, iron deficiency promoted the colonization and proliferation of *Salmonella* in mice [41], while high intraperitoneal iron promoted rapid proliferation of *E. coli* and caused death of neonatal pigs [10]. Infants have immature mucosal immunity and are susceptible to disruption of intestinal immune homeostasis at weaning leading to pathogen infection [42]. Intestinal macrophages are the first line of intestinal defense against pathogens and have a key role in maintaining intestinal homeostasis. Inhibition of macrophage polarization to CD86^+^ subpopulation and promotion of CD206^+^ subpopulation polarization can alleviate intestinal inflammation [43]. In our study, iron overload reduced the population of CD86^+^ subpopulation, and different iron levels did not significantly affect CD206^+^ subpopulation in jejunum. Studies have reported that iron overload reduced NF-κB p65 nuclear translocation in bone marrow macrophages, thereby decreasing the population of cells expressing M1 co-stimulatory CD86 and MHC-II molecules [44]. However, in another study, iron overload induced M1 polarization by increasing ROS production and inducing p53 acetylation in RAW 264.7 macrophages [45]. The inconsistent results may be due to the duration of iron treatment time and the tissue specificity of macrophages [46].

T cell differentiation also influences neonatal intestinal mucosal immune development [47]. In our study, the percentages of CD45^+^ and CD3^+^ T cells in the jejunum of iron-deficient and iron-overloaded pigs were decreased, suggesting that iron-deficiency and iron-overload led to intestinal immunodeficiency. Differentiation of intestinal T lymphocytes, especially Tregs and their key cytokine IL-10, contribute to the maintenance of Lgr5^+^ ISC in the small intestine and promote intestinal epithelial cell differentiation [48]. Thus, intestinal T-cell differentiation helps maintain the intestinal barrier against pathogens. In a recent study, single-cell RNA sequencing revealed an increased proportion of cytotoxic T lymphocytes in ileum of weaned pigs. In addition, this study found that the differentiation of cytotoxic CD8^+^ T cells induced mitochondrial dysfunction promoting epithelial cell apoptosis by promoting granzyme B (*GZMB*) expression, which ultimately led to severe intestinal inflammation [49]. Similar to this results, iron deficiency and iron overload promoted the differentiation of CD8^+^ cytotoxic T cells in the jejunum and MLN of pigs, which may disrupt intestinal immune homeostasis. In DSS-induced colitis mice (6–8 weeks), iron deficiency promoted colonic CD4^+^ T cell infiltration [50]. However, iron deficiency and iron overload had no significant effect on CD4^+^ T cells in jejunum and MLN in this experiment. This may be due to the fact that neonatal CD4^+^ T cells remain immature throughout the postnatal period, contributing to the prevention of self-reactivity, maintenance of an extensive TCR pool and establishment of lifelong immune homeostasis [51].

## Conclusion

In summary, our results demonstrated that iron deficiency and iron overload led to downregulation of intestinal barrier and antioxidant marker genes in jejunum and promoted CD8^+^ T cell differentiation in the jejunum and MLN of pigs, disrupting gut health. Furthermore, iron deficiency and iron overload disrupted redox homeostasis and affected host innate and adaptive immunity. These findings emphasized the importance of iron in regulating neonatal immunity and development, and confirmed that supplementation of 200 mg FeDex to neonatal pigs is justified. The present study will provide important clues for further investigation on the interactions between iron and immune system.

## Data Availability

The datasets used during the current study are available from the corresponding author on reasonable request.

## Abbreviations

Bas#: Basophil count
CAT: Catalase
DSS: Dextran sulfate sodium salt
*E. coli*: *Escherichia coli*
Eos#: Eosinophil count
GZMB: Granzyme B
GSH-Px: Glutathione peroxidase
HGB: Hemoglobin
HCT: Erythrocyte pressure volume
IDA: Iron deficiency anemia
Ig: Immunoglobulin
IFN-γ: Interferon-γ
IL: Interleukin
Lym#: Lymphocyte count
MLN: Mesenteric lymph nodes
MCV: Mean corpuscular volume
MCH: Mean corpuscular hemoglobin
MCHC: Mean corpuscular hemoglobin concentration
MDA: Malondialdehyde
Mon#: Monocyte count
Neu#: Neutrophil count
PEDV: Porcine epidemic diarrhea virus
PLT: Platelet
PCT: Platelet hematocrit
RBC: Red blood cell
TIBC: Total iron binding capacity
TS: Transferrin saturation
T-SOD: Total superoxide dismutase
T-AOC: Total antioxidant capacity
TNF-α: Tumor necrosis factor-α
UIBC: Unbound iron binding capacity
WBC: White blood cells
